# Diabetic Cardiomyopathy Uncovered: Transcriptomics, NLRP3, and Carvedilol Mechanisms

**DOI:** 10.1155/2024/9378405

**Published:** 2024-11-23

**Authors:** Alimujiang Abudoureyimu, Alimu Aihaiti, Nuliman Abudoujilili, Mayila Tuergong, Guzainuer Adili, Maihebubaimu Maimaiti, Dilinuer Mohetaer, Yimamumaimaiti Maiamaitishawuti

**Affiliations:** Department of Cardiology, The First People's Hospital of Kashi Prefecture, Kashi, China

**Keywords:** carvedilol, diabetic cardiomyopathy, H9C2 cardiomyocytes, NLRP3 inflammasome, pyroptosis, ROS inhibition

## Abstract

**Background:** This study investigates the impact of a high-sugar environment on H9C2 cardiomyocytes and explores the protective effects of carvedilol in the context of diabetic cardiomyopathy (Dia-CM). Transcriptomic analysis identified 21,655 differentially expressed genes associated with Dia-CM, demonstrating significant separation among samples.

**Methods:** H9C2 cardiomyocytes were cultured in a high-sugar environment to simulate Dia-CM conditions. Cell viability, cytokine levels, and protein expression were assessed using CCK-8 assays, ELISA, and Western blot techniques. Intervention experiments with NLRP3, caspase-1, and ROS inhibitors were conducted to evaluate their protective effects. The therapeutic potential of carvedilol was assessed by examining its impact on cell viability, cytokine levels, and key biomarkers. An in-depth analysis of carvedilol's regulatory effects on ROS and key proteins in H9C2 cells was also conducted.

**Results:** In vitro, a high-sugar environment significantly reduced H9C2 cell survival, increased ROS levels, activated inflammatory responses, and upregulated NLRP3, caspase-1, and GSDMD-N proteins. Inhibitors of NLRP3, caspase-1, and ROS ameliorated these effects. Carvedilol treatment improved cell activity, reduced inflammatory cytokine levels, suppressed ROS production, and downregulated NLRP3, pro-caspase-1, GSDMD-N, and p-NF-*κ*B proteins. Moderate-dose carvedilol exhibited optimal intervention effects.

**Conclusions:** A high-sugar environment induces cardiomyocyte damage through ROS production and NLRP3 inflammasome activation. Inhibitors of NLRP3, caspase-1, and ROS provide effective protection. Carvedilol significantly mitigates the detrimental effects of a high-sugar environment on H9C2 cardiomyocytes, potentially through inhibiting the NLRP3-ASC inflammasome and caspase-1/GSDMD-dependent signaling pathway-mediated pyroptosis. These findings offer insights into Dia-CM mechanisms and highlight carvedilol as a promising therapeutic intervention.

## 1. Introduction

Diabetic cardiomyopathy (Dia-CM) is a diabetes-related cardiac disease characterized by structural and functional changes in the myocardium, independent of the presence of coronary artery disease, hypertension, or other cardiac conditions [1]. With the increasing number of diabetic patients, Dia-CM has become a significant cause of cardiovascular disease and mortality worldwide [2]. The pathophysiological mechanisms of Dia-CM are complex and involve various cellular and molecular pathways, with the exact mechanisms still not fully understood. A high glucose (HG) environment is considered a key driving factor in Dia-CM development [3], leading to impaired myocardial cell function, increased oxidative stress, and inflammation [4–6]. Therefore, investigating the pathogenesis of Dia-CM and potential therapeutic drugs is an urgent issue.

In a HG environment, myocardial cells undergo a series of detrimental changes, including apoptosis, autophagy, and increased oxidative stress [7–9]. These changes result in myocardial fibrosis, ventricular remodeling, and decreased cardiac function, ultimately leading to heart failure [10]. Studies have shown that inflammatory responses play a critical role in this process, particularly the activation of the nucleotide-binding oligomerisation domain-like receptor family pyrin domain-containing protein 3 (NLRP3) inflammasome [11, 12].

The NLRP3 inflammasome plays a crucial role in HG and dyslipidemia [13, 14]. This mechanism promotes the cleavage of gasdermin D (GSDMD), leading to the generation of lipophilic GSDMD-N and subsequent cell fusion [15, 16]. However, it also promotes the secretion of interleukin-1*β* (IL-1*β*) and IL-18, participating in the regulation of inflammation [17]. The NLRP3 inflammasome is closely associated with dilated cardiomyopathy (Dia-CM) [18]. Studies have revealed significantly upregulated expression of NLRP3 and caspase-1 in the myocardium of Dia-CM rats. Inhibition of NLRP3 channels or caspase-1 enzyme reduces myocardial fibrosis and significantly improves cardiac function in Dia-CM rats [19, 20]. All these studies indicate that activation or pyroptosis of the NLRP3 inflammasome could be a promising therapeutic strategy for Dia-CM.

Carvedilol is a nonselective betablocker that has been used in the treatment of hypertension, coronary artery disease, and heart failure [21]. In recent years, research has revealed that carvedilol also possesses antioxidant, anti-inflammatory, and cytoprotective properties [22, 23]. Considering these characteristics, carvedilol may alleviate myocardial cell damage in a HG environment by inhibiting NLRP3 inflammasome activation.

Based on the above, the aim of this study is to investigate the mechanisms of cardiac cell injury in a HG environment and evaluate the protective effect of carvedilol on H9C2 myocardial cells, particularly its impact on NLRP3 inflammasome activity.

## 2. Materials and Methods

### 2.1. Transcriptome Analysis of Human-Induced Pluripotent Stem Cell–Derived Cardiomyocytes in Dia-CM

Transcriptomic RNA sequencing data related to diabetic cardiomyopathy were downloaded from the Gene Expression Omnibus (GEO) database (http://www.ncbi.nlm.nih.gov/geo/), specifically the GSE62203 chip dataset. The dataset consisted of a total of eight samples, including four control samples (normal-cultured human-induced pluripotent stem cell–derived cardiomyocytes) and four model samples (human-induced pluripotent stem cell–derived cardiomyocytes exposed to glucose, endothelin-1, and cortisol, representing the phenotype of diabetic cardiomyopathy).

### 2.2. Differential Gene Analysis

Differential gene expression analysis was performed using the GEO dataset. Genes were selected based on the criteria of |logFC| > 1 and *p* < 0.05. The R package limma (https://www.bioconductor.org/packages/release/bioc/html/limma.html) was utilized for this purpose. Heatmaps and volcano plots were generated using the R packages pheatmap (https://cran.r-project.org/web/packages/pheatmap/index.html) and ggplot2 (https://cran.r-project.org/web/packages/ggplot2/index.html), respectively. All analyses were conducted using R Version 4.2.1 (R Foundation for Statistical Computing).

### 2.3. Functional Enrichment Analysis

Functional enrichment analysis was performed based on differentially expressed genes using the GO and KEGG databases in combination with log fold change (logFC). The R software packages “clusterProfiler [4.4.4]” and “GOplot [1.0.2]” were utilized for this analysis. The GO enrichment analysis included three components: biological process (BP), cellular component (CC), and molecular function (MF), with a significance threshold of *p* < 0.05. Enriched pathways and visualizations of the associated genes were conducted. KEGG enrichment analysis was also performed with a significance threshold of *p* < 0.05, again followed by pathway enrichment and gene visualization. By comparing the annotated genes and the enriched pathways, key mechanisms related to diabetic cardiomyopathy were identified.

### 2.4. Cell Culture

In our study, H9C2 rat cardiomyocytes were selected as the experimental model, which was purchased from Procell Life Science & Technology Co., Ltd. (CL-0089). The cells were cultured using modified Dulbecco's Modified Eagle's Medium (DMEM, 41965120, Gibco) supplemented with HG. The culture medium was also supplemented with 10% fetal bovine serum (FBS, 10270106, Gibco) and 1% penicillin/streptomycin (15140148, Gibco) to provide the necessary nutrients and antibiotic protection for cell growth. After routine digestion, subculture, and cryopreservation, all cells were cultured at 37°C, 5% CO_2_, and saturated humidity.

MCC950 was acquired from MedChemExpress (catalog number: HY-12815). It was dissolved in dimethyl sulfoxide (DMSO) as per the instructions and stored at −80°C, then used to treat cells at a concentration of 200 ng/mL for 24 h before subsequent experiments. H9C2 cells were pretreated with 10 *μ*M MCC950 for 2 h before being transferred to a DMEM culture medium containing 35 mM glucose for an additional 24 h.

VX-765 was obtained from MedChemExpress (catalog number: HY-13205) and prepared by dissolving in DMSO following the instructions, then stored at −80°C. H9C2 cells received a 2-h pretreatment with 20 *μ*M VX-765 before being transferred to a DMEM culture medium containing 35 mM glucose and further incubated for 24 h.

N-Acetyl-L-cysteine (NAC) was purchased from MedChemExpress (catalog number: HY-134495). NAC was dissolved in DMSO according to the instructions and stored at −80°C. H9C2 cells were pretreated with 50 *μ*M NAC for 2 h before being transferred to a DMEM culture medium containing 35 mM glucose for an additional 24 h.

The cell groups were categorized as follows: (1) blank group (control group): H9C2 cells were cultured in DMEM containing 5.5 mM glucose for 24 h. (2) Model group (HG): H9C2 cells were incubated in DMEM containing 35 mM glucose for 24 h to establish a HG-induced cell model. (3) Model+MCC950 intervention group (HG+MCC950): a cell model induced by HG was established after a pretreatment with 10 *μ*M MCC950. (4) Model+VX-765 intervention group (HG+VX-765): a cell model induced by HG was created after treatment with 20 *μ*M VX-765. (5) NAC intervention group (HG+NAC): a cell model induced by HG was established after treatment with 50 *μ*M NAC.

### 2.5. Carvedilol Concentration Screening

To examine the effect of carvedilol (HY-B0006, MedChemExpress) on H9C2 rat cardiomyocytes at different concentrations, we divided the cells into five groups and cultured them in DMEM containing 5.5 mM glucose for 24 h as preparation for the experiments.

(1) Control group: H9C2 cells continued to be cultured in DMEM containing 5.5 mM glucose for 24 h. (2) HG model group (HG): H9C2 cells were cultured in DMEM containing 35 mM glucose for 24 h to establish a cell model induced by HG. (3) Model+low-dose carvedilol intervention group (HG+CAR-low): H9C2 cells were first cultured in DMEM containing 35 mM glucose, followed by the addition of 5 *μ*M carvedilol for a total duration of 24 h. (4) Model+medium-dose carvedilol intervention group (HG+CAR-mid): H9C2 cells were cultured in DMEM containing 35 mM glucose and 10 *μ*M carvedilol for 24 h. (5) Model+high-dose carvedilol intervention group (HG+CAR-high): H9C2 cells were cultured in DMEM containing 35 mM glucose and 15 *μ*M carvedilol for 24 h. After experimental verification, the most suitable concentration was determined, and the same method was used for further mechanism validation.

To further investigate the synergistic effects of carvedilol with other compounds, we divided the cells into six groups: (1) control group (blank): H9C2 cells were cultured in DMEM containing 5.5 mM glucose for 24 h. (2) Model group (HG): H9C2 cells were cultured in DMEM containing 35 mM glucose for 24 h to establish a HG-induced cell model. (3) Model+CAR group (HG+CAR): H9C2 cells were cultured in DMEM containing 35 mM glucose and 10 *μ*M carvedilol for 24 h. (4) Model+MCC950 intervention group (HG+MCC950): H9C2 cells were pretreated with 10 *μ*M MCC950 for 2 h and then cultured in DMEM containing 35 mM glucose and 10 *μ*M carvedilol for 24 h. (5) Model+VX-765 intervention group (HG+VX-765): H9C2 cells were pretreated with 20 *μ*M VX-765 for 2 h and then cultured in DMEM containing 35 mM glucose and 10 *μ*M carvedilol for 24 h. (6) NAC intervention group (HG+NAC): H9C2 cells were pretreated with 50 *μ*M NAC for 2 h and then cultured in DMEM containing 35 mM glucose and 10 *μ*M carvedilol for 24 h.

### 2.6. CCK-8

To accurately assess the proliferative capacity of H9C2 myocardial cells under different experimental conditions, we utilized the CCK-8 method for detection. Initially, we collected H9C2 cells grown to 90% density through trypsin digestion and adjusted them to a concentration of 5 × 104 cells/mL, forming a single-cell suspension. Subsequently, we transferred 100 *μ*L of the cell suspension to a 96-well plate, with each well serving as a single sample, resulting in five replicate wells to enhance experimental accuracy.

Following 24 h of adherence culture, we discarded the culture medium and intervened with specific drugs or conditions according to the predetermined experimental groups. After the intervention period, the supernatant was removed, and 100 *μ*L of a pre-prepared solution of the CCK-8 cell counting kit (C0038, Beyotime) was mixed with the cells in each well. This mixture was then incubated at 37°C for 1 h to allow the CCK-8 reagent to react with viable cells, resulting in the generation of a yellow formazan product. Finally, we used a microplate reader to measure the optical density (OD value) of each well at a wavelength of 450 nm. The magnitude of the OD value reflects the quantity of viable cells in the well and thus represents the proliferative capacity of the cells indirectly.

### 2.7. Lactate Dehydrogenase (LDH) Activity Assay

To assess the extent of damage to H9C2 myocardial cells under different experimental conditions, we analyzed the activity of LDH. LDH is an enzyme widely present in cells, and its activity increases when cell membranes are damaged and released into the culture medium, making it a commonly used marker for evaluating cellular injury. After completing the experimental groups and drug interventions, we collected the supernatant of cell culture for LDH activity measurement. The specific steps were as follows: we used the LDH assay kit (C0016, Beyotime) and prepared all necessary reagents and reaction solutions according to the manufacturer's instructions. The supernatant was added to a 96-well plate containing LDH reaction solution, with an appropriate amount of samples added to each well to ensure sufficient replicate wells for increased data accuracy. The 96-well plate was incubated at 37°C for a specified time to allow sufficient reaction between LDH and the substrate. After the reaction, the OD value was measured at a specified wavelength using a microplate reader. LDH concentration for each sample was calculated using the following formula: LDH concentration (U/L) = (actual OD value − control OD value)/(standard OD value − blank OD value) × standard concentration (0.2 *μ*mol/L) × 1000.

### 2.8. Quantitative Analysis of IL-1*β* and IL-18 Levels

To quantitatively analyze the levels of IL-1*β* and IL-18 secreted by cells, as well as their proportion in total cellular protein, we employed the enzyme-linked immunosorbent assay (ELISA) and protein content determination methods. The specific steps were as follows: cell culture and treatment: firstly, H9C2 cardiomyocytes were seeded into 12-well cell culture plates. After 24 h of intervention, we collected the cell culture medium for subsequent IL-1*β* and IL-18 content determination. IL-1*β* and IL-18 levels in the medium were measured using the IL-1*β* and IL-18 ELISA kits (Jiangsu Kote Biological Technology Company), following the instructions. Specifically, standard samples and test samples were added to microplates precoated with IL-1*β* or IL-18 antibodies. After incubation and washing, detection antibodies and substrates were added, and absorbance values were read using a microplate reader at 450 nm wavelength. The concentrations of IL-1*β* and IL-18 in the samples were calculated based on the standard curve. Simultaneously, cells were harvested, and cell lysis was prepared using a method involving double-distilled water and hydrochloric acid. The protein content in the cell lysate was determined using the BCA protein concentration determination kit (P0010, Beyotime), according to the instructions. Finally, the ratio of IL-1*β* and IL-18 content to total protein content was calculated to evaluate their relative levels within the cells.

### 2.9. Reactive Oxygen Species Level Detection

To accurately assess the variation in ROS levels in H9C2 cardiomyocytes under different experimental conditions, we employed flow cytometry in combination with the fluorescent probe DCFH-DA. After digestion with trypsin and adherent culture, cells were seeded in a 6-well plate at a concentration of 5 × 10^4^ cells/mL. Each well was supplemented with 500 *μ*L of cell suspension and incubated at 37°C, 5% CO_2_ for 24 h. Subsequently, intervention treatments were conducted according to the experimental groups, with three replicate wells per group. After the intervention, each well was supplemented with 2 mL of PBS containing 10 *μ*M DCFH-DA (S0033S, Beyotime) and incubated at 37°C for 30 min, allowing DCFH-DA to enter the cells and react with ROS, producing fluorescence. Following centrifugation and PBS washing, the fluorescence intensity within the cells was detected using a flow cytometer with an excitation wavelength of 500 nm and an emission wavelength of 525 nm. Through this series of procedures, we were able to accurately and effectively evaluate the ROS levels in H9C2 cardiomyocytes under different experimental conditions, providing robust data support for further research on the role of ROS in myocardial cell function and damage.

### 2.10. Immunoblotting

First, H9C2 cardiomyocytes were plated in a 12-well plate, and after 24 h of treatment, cell lysis buffer (P0013B, Beyotime) was used to collect and lyse the cells, releasing intracellular proteins. Subsequently, proteins were separated by SDS-PAGE electrophoresis and transferred onto a polyvinylidene difluoride (PVDF) membrane (FFP26, Beyotime) using a method called electrotransfer. Next, we blocked nonspecific binding sites on the membrane with 5% skim milk to reduce background signals. The membrane was then incubated overnight with specific primary antibodies against NLRP3 (ab263899, 1:1000, Abcam), ASC (ab309497, 1:1000, Abcam), pro-caspase-1 (ab179515, 1:1000, Abcam), GSDMD-N (39754, 1:1000, CST), nuclear factor-kappa B (NF-*κ*B) (8242, 1:1000, CST), phosphorylated NF-*κ*B (p-NF-*κ*B) (3033, 1:1000, CST), and *β*-actin (4967, 1:1000, CST) to ensure optimal binding. The following day, the membrane was incubated with secondary antibodies anti-rabbit (ab150077, 1: 5000, Abcam) or anti-mouse (ab150113, 1:5000, Abcam), which were conjugated with horseradish peroxidase (HRP), enabling detection through enhanced chemiluminescence (ECL). Finally, protein bands were visualized using a gel imaging system, and the band intensities were quantitatively analyzed using ImageJ to assess the expression levels of the target proteins.

### 2.11. Statistical Analysis

In this biomedical research, all collected data were presented in the form of means ± standard deviations, and statistical analysis was performed using SPSS 24.0 software. Firstly, normality testing and homogeneity of variance testing were conducted on the data to ensure that they meet the assumptions for one-way analysis of variance (ANOVA). When the data were confirmed to follow a normal distribution and exhibit homogeneity of variance, an ANOVA was performed to compare the average differences between different experimental groups. If the ANOVA results showed a *p* value less than 0.05, it was considered a statistically significant difference among the experimental groups. Additionally, multiple comparison methods, such as Tukey's HSD and Bonferroni's correction, were applied to determine the specific group differences that are statistically significant. Finally, the data were interpreted in detail based on the results of the statistical analysis and presented in clear tables and figures in the research report to ensure transparency and interpretability of the findings.

## 3. Results

### 3.1. Transcriptomics Reveal Key Roles of Inflammation and Apoptosis in the Pathogenesis of Diabetic Cardiomyopathy

**Figure 1:**
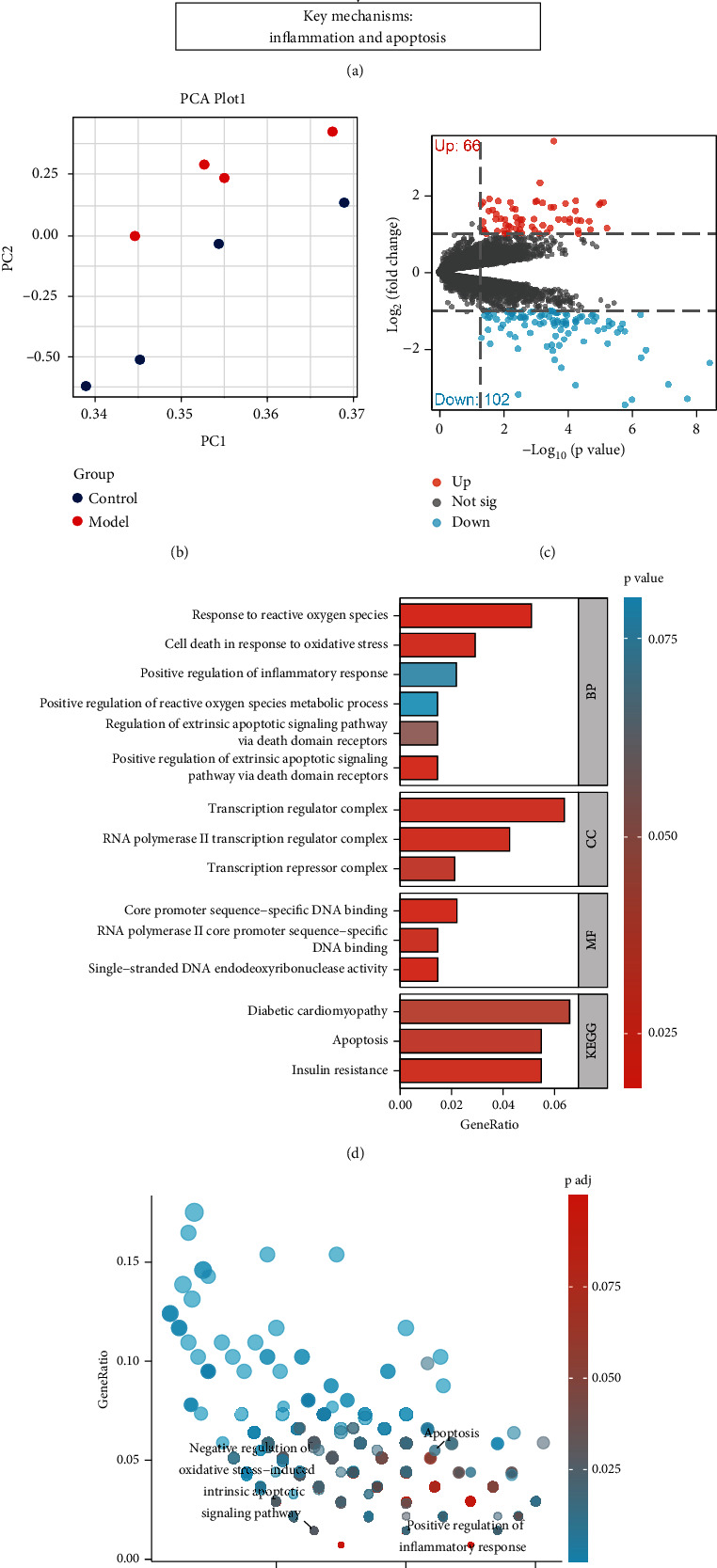
Principal component analysis and functional annotation of differentially expressed genes in diabetic cardiomyopathy. Note: (a) Schematic diagram of the transcriptomic screening process. (b) Principal component analysis (PCA) shows the separation of different groups of samples based on gene expression levels. (c) Volcano plot of differentially expressed genes. The *x*-axis represents the logarithmic fold change (logFC), and the *y*-axis represents the negative logarithm of the *p* value (−log10 *p* value). Red dots represent upregulated genes, blue dots represent downregulated genes, and gray dots represent genes with no significant difference. Each dot represents a sample, and different colors represent different groups. A threshold of |logFC| > 1 and *p* < 0.05 was set for significant differential expression. (d, e) Results of GO functional analysis and KEGG pathway analysis.

Diabetic cardiomyopathy (Dia-CM) is a heart disease associated with diabetes, and its occurrence and progression are related to various molecular mechanisms in which inflammation and cellular apoptosis play crucial roles. To further investigate the specific roles of these two BPs in Dia-CM, we downloaded relevant expression profiles from the GEO database and performed a series of systematic transcriptomic analyses (Figure 1(a)).

Using the limma package, we successfully identified 21,655 differentially expressed genes (Table 1). This large number of differentially expressed genes provided us with a comprehensive landscape of gene expression changes and laid a solid foundation for further investigation. To validate whether these differentially expressed genes exhibit consistent trends among samples, we conducted principal component analysis (PCA), which showed a clear separation of samples from different groups based on gene expression (Figure 1(b)). This not only enhanced the credibility of our dataset results but also suggested the presence of underlying biological differences awaiting further exploration.

To more precisely identify key genes associated with the occurrence and progression of Dia-CM, we further set the criteria |logFC| > 1 and *p* < 0.05. Compared to the control group, the model group had 102 genes with downregulated expression and 66 genes with upregulated expression (Figure 1(c) and Table 2). GO functional analysis and KEGG pathway analysis of these 168 differentially expressed genes revealed that in terms of BPs, these genes were primarily enriched in processes related to ROS, extrinsic apoptosis signaling regulated by the death domain receptor, and positive regulation of inflammatory response. In terms of CCs, the differentially expressed genes were enriched in sites such as the RNA Polymerase II transcription regulation complex, transcription regulatory complex, and transcriptional repressor complex. In terms of MFs, enrichment was observed in activities such as single-stranded DNA endonuclease activity, DNA binding specific to core promoter sequences, and RNA Polymerase II core promoter sequence-specific DNA binding (Figures 1(d) and 1(e)). Furthermore, the KEGG pathway analysis revealed that the differentially expressed genes were mainly enriched in signaling pathways such as insulin resistance, cellular apoptosis, and diabetic cardiomyopathy (Figures 1(d) and 1(e)).

In conclusion, through in-depth transcriptomic analysis, we have revealed the crucial roles of inflammation and apoptosis processes in the occurrence and progression of Dia-CM. Further research is needed to elucidate the specific mechanisms underlying these processes.

### 3.2. The Protective Effect of NLRP3 Inflammasome Activation and Its Inhibitors on H9C2 Cardiomyocytes in a HG Environment

**Figure 2:**
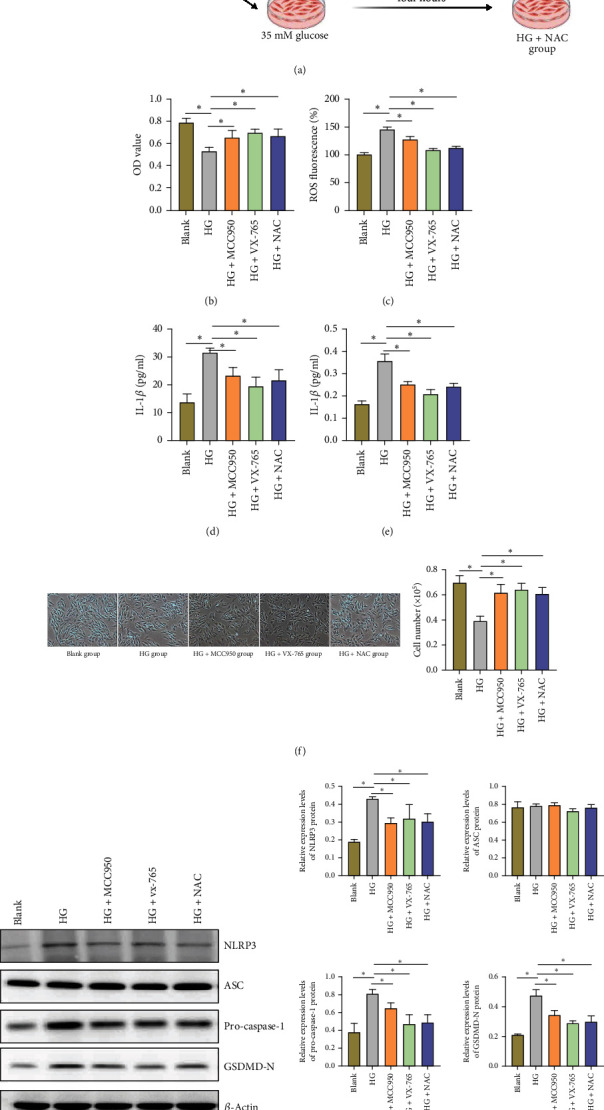
Effects of NLRP3 inflammasome activation and its inhibitors on H9C2 cardiomyocytes under high glucose conditions. Note: (a) Schematic diagram of the experimental procedure investigating the regulation of inflammation and apoptosis on cardiomyocytes under high glucose conditions. (b) CCK-8 assay comparing the cell viability under different treatment conditions (blank group, HG group, HG+MCC950 intervention group, HG+VX-765 intervention group, and HG+NAC intervention group). (c) Comparison of ROS levels in cells under different treatment conditions. (d, e) ELISA detection of levels of inflammatory cytokines IL-1*β* and IL-18 in cells under different treatment conditions. (f) Schematic diagram of cell culture under different treatment conditions (×100). (g, h) Western blot analysis of protein expression levels of NLRP3, caspase-1, ASC, and GSDMD-N in cells under different treatment conditions and their quantitative analysis. (i) Brief schematic diagram of the regulation of inflammation and apoptosis on cardiomyocytes under high glucose conditions. The statistical analysis employed one-way analysis of variance (ANOVA), followed by Tukey's multiple comparison test; ⁣^∗^*p* < 0.05.

One of the key factors in the pathological process of diabetic cardiomyopathy (Dia-CM) is the activation of cardiomyocyte damage and inflammatory response under HG conditions. As a pattern recognition receptor, NLRP3 can sense both intracellular and extracellular stress signals, including an increase in ROS. ROS not only activates NLRP3 but also directly causes cell damage. The activation of NLRP3 triggers the aggregation of ASC and recruits and activates caspase-1 through the CARD domain, thereby promoting the maturation and release of inflammatory cytokines and triggering an inflammatory response. In some cases, excessive activation of caspase-1 can also lead to pyroptosis, a process associated with programmed cell death, leading to cardiomyocyte death. This series of events may result in cardiac inflammation, damage, and functional deterioration, which are associated with the development of various heart diseases. Therefore, the balance and regulation of NLRP3, caspase-1, ASC, and ROS in cardiomyocytes are crucial for maintaining heart health. Therefore, we chose H9C2 cardiomyocytes as an experimental model to investigate their interactions under HG conditions (Figure 2(a)).

In the CCK-8 experiment, we observed a significant decrease in the survival rate of H9C2 cells in the HG treatment group compared to the blank group. This result suggests that a HG environment has a pronounced inhibitory effect on the survival of cardiomyocytes. Further experiments showed that by applying NLRP3 inhibitor (MCC950), caspase-1 inhibitor (VX-765), and ROS inhibitor (NAC), we were able to significantly improve the viability of H9C2 cells under HG conditions (Figures 2(b) and 2(f) and Table 1), indicating that these inhibitors can effectively intervene in cell damage caused by HG.

The determination of ROS levels showed a significant increase in ROS levels in H9C2 cells under HG treatment, while the treatment with MCC950, VX-765, and NAC significantly reduced ROS levels (Figure 2(c) and Table 2). These results suggest a close correlation between the increase in ROS and the damage to cardiomyocytes under HG conditions, and inhibiting ROS production helps alleviate cell damage.

ELISA detection further revealed the activation of inflammatory responses under HG conditions. We found that compared to the blank group, the levels of IL-1*β* and IL-18 in H9C2 cells in the HG group significantly increased, while these levels were significantly reduced after treatment with MCC950, VX-765, and NAC (Figures 2(d), 2(e), and 2(f) and Table 2).

Western blot analysis showed that HG treatment significantly upregulated the expression of NLRP3, caspase-1, and GSDMD-N in H9C2 cells, while the expression of apoptosis-associated speck-like protein containing a CARD (ASC) remained unchanged. Pretreatment with MCC950, VX-765, and NAC effectively reduced the expression of NLRP3, caspase-1, and GSDMD-N but had no effect on ASC expression (Figures 2(g) and 2(h) and Table 3).

In conclusion, our study results demonstrate that a HG environment can induce damage to H9C2 cardiomyocytes, increase ROS production, activate inflammatory responses, and promote excessive expression of NLRP3 inflammasome-related proteins (Figure 2(i)). However, inhibitors of NLRP3, caspase-1, and ROS can effectively intervene in these adverse effects and provide protection to cardiomyocytes.

### 3.3. Regulation of the Biological Effects of Carvedilol on HG-Treated H9C2 Cells and Optimal Concentration Screening

In order to explore potential intervention strategies to alleviate the adverse effects of a HG environment on H9C2 myocardial cells, this study evaluated the therapeutic effect of carvedilol and its effects on cell viability and key biomarkers at different concentrations.

**Figure 3:**
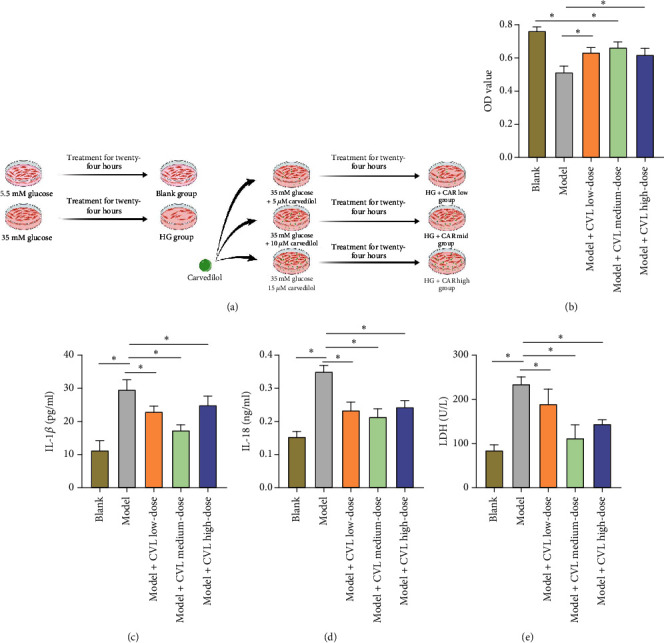
Regulation of high glucose-treated H9C2 cell biological effects and optimal concentration screening by carvedilol. Note: (a) Schematic diagram of the experimental procedure investigating the regulatory effects of carvedilol at concentrations of 5, 10, and 15 *μ*M on the biological effects of high glucose-treated H9C2 cells. (b) Effects of different concentrations of carvedilol on cell viability under high glucose conditions. Cell viability was assessed using the CCK-8 assay to quantify cell survival rates. (c, d) Effects of different concentrations of carvedilol on IL-1*β* and IL-18 levels in the supernatant of high glucose-treated H9C2 cells, as detected by ELISA. (e) Evaluation of the effects of different concentrations of carvedilol on cell membrane integrity of high glucose-treated H9C2 cells using LDH release assay. The statistical analysis employed one-way analysis of variance (ANOVA), followed by Tukey's multiple comparison test; ⁣^∗^*p* < 0.05.

Firstly, it was observed that treatment with carvedilol significantly improved the activity of H9C2 cells cultured in a HG environment, with the medium dosage of carvedilol showing the best effect (Table 4 and Figure 3(a)). Additionally, carvedilol treatment was found to significantly decrease the levels of IL-1*β* and IL-18 in the supernatant of HG group H9C2 cells. Similarly, the medium dosage treatment displayed the most favorable outcome (Table 4 and Figures 3(b) and 3(c)). Moreover, it was noted that the levels of LDH in the supernatant of HG group H9C2 cells were significantly higher than those in the blank group. After carvedilol treatment, the levels of LDH were significantly reduced, with the medium dosage achieving the best outcome (Table 4 and Figures 3(d) and 3(e)).

In conclusion, carvedilol exhibits significant regulatory effects and effectively improves the adverse biological effects of a HG environment on H9C2 myocardial cells. The medium dosage of carvedilol demonstrates the optimal intervention effect, providing important references for subsequent drug screening and treatment.

### 3.4. The Effects of Carvedilol on the Expression of Key Proteins and ROS Levels in H9C2 Cells Induced by HG

With a deeper understanding of the mechanisms of myocardial cell injury in a HG environment, the roles of ROS and various key proteins in cell damage have garnered increasing attention. In order to evaluate the therapeutic effects of carvedilol, this study conducted a comprehensive analysis of its impact on ROS levels and the expression of key proteins in H9C2 cells.

**Figure 4:**
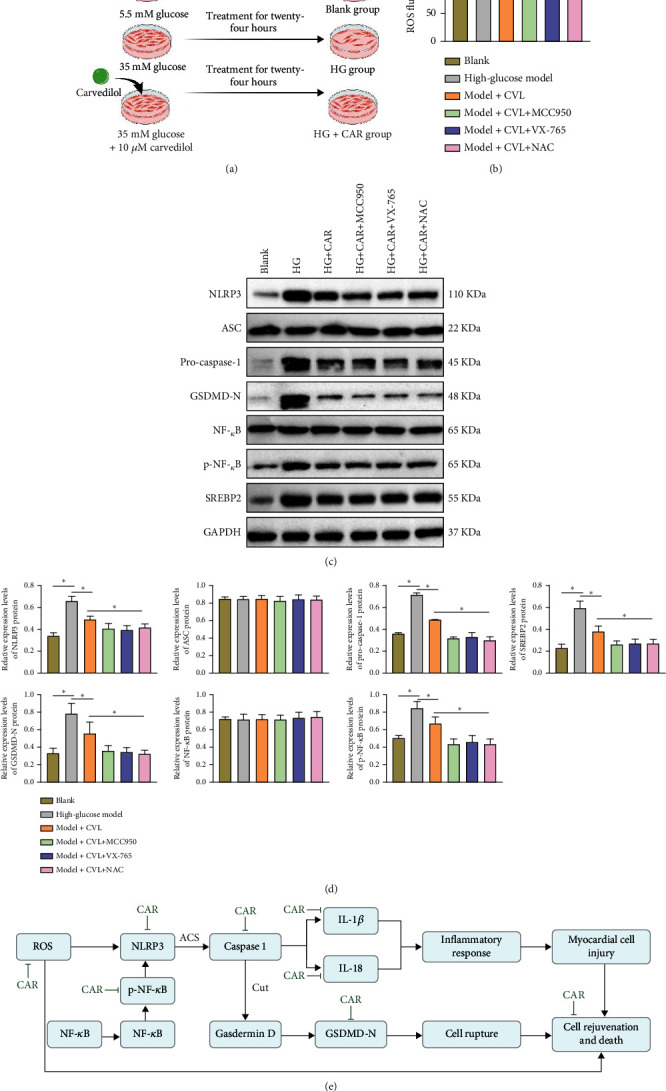
Regulation of ROS levels and key protein expression in high glucose-treated H9C2 cells by carvedilol. Note: (a) Schematic diagram of the experimental procedure investigating the effects of carvedilol at a concentration of 10 *μ*M on key mechanisms in high glucose-treated H9C2 cells. (b) Effects of carvedilol on ROS levels in H9C2 cardiomyocytes under high glucose conditions. (c, d) Western blot analysis showing the effects of carvedilol on the expression of key proteins such as NLRP3, pro-caspase-1, GSDMD-N, SREBP2, NF-*κ*B, and p-NF-*κ*B in high glucose-treated H9C2 cells. (e) Brief schematic diagram of carvedilol's regulation of inflammation and apoptosis in cardiomyocytes under high glucose conditions. Statistical analysis involved one-way analysis of variance (ANOVA) followed by Tukey's post hoc test, where significance was set at *p* < 0.05.

Initially, we observed that carvedilol intervention significantly reduced the levels of ROS in HG group H9C2 cells, providing a relatively stable environment for the cells. Moreover, the addition of the NLRP3 inhibitor (MCC950), caspase-1 inhibitor (VX-765), or ROS inhibitor (NAC) further decreased the ROS levels in H9C2 cells (Table 5 and Figure 4(b)). Further analysis revealed that in the HG environment, the protein expression levels of NLRP3, pro-caspase-1, GSDMD-N, SREBP2, nuclear factor-kappa B (NF-*κ*B), and phosphorylated NF-*κ*B (p-NF-*κ*B) in H9C2 cells were significantly higher compared to the blank group. However, after carvedilol treatment, the expression of these key proteins was significantly suppressed. When comparing HG+CAR with HG+CAR+MCC950, HG+CAR+VX-765, and HG+CAR+NAC groups, the protein expression levels of NLRP3, pro-caspase-1, and GSDMD-N were further significantly inhibited, while SREBP2, NF-*κ*B, and p-NF-*κ*B showed no significant change in expression levels (Table 6 and Figures 4(b) and 4(c)).

In summary, carvedilol not only significantly reduces ROS levels in H9C2 cells under HG conditions but also effectively inhibits the overexpression of multiple key proteins. The underlying molecular mechanisms are depicted in Figure 4(e), providing a solid theoretical basis for further medical interventions in HG-induced myocardial cell damage.

## 4. Discussion

Diabetic cardiomyopathy (Dia-CM) is a common complication of diabetes, and it can lead to heart failure, arrhythmias, and sudden cardiac death [24]. It involves extensive local myocardial damage and necrosis due to metabolic disturbances in the myocardium and microvascular disease in the heart caused by diabetes [25, 26]. In the field of cardiovascular disease, there has been extensive research on the dysfunction of cardiac cells under HG conditions [27]. Studies have shown that HG conditions significantly impair cardiac cells and their function [28, 29]. However, the mechanism of cardiac cell damage remains unclear.

Through transcriptomic analysis, we discovered that inflammation and pyroptosis play a crucial role in Dia-CM. Pyroptosis is an inherent inflammatory process characterized by rapid cell membrane rupture and release of pro-inflammatory cytokines [30]. Previous studies have shown that pyroptosis is involved in the progression of cardiac diseases [31, 32]. In patients with Dia-CM, the extent of pyroptosis in cardiac cells is significantly increased and upregulated in human ventricular myocardial cells under HG conditions [33, 34]. Carvedilol has antipyroptotic effects and can protect against peritonitis by inhibiting the activation of NLRP3 inflammasomes and macrophage pyroptosis [35, 36]. In this study, we stimulated H9C2 cells with 35 mM glucose to establish a model of myocardial cell injury and used carvedilol as an intervention to determine if HG-induced myocardial cell damage is related to pyroptosis. Initially, we cultured rat myocardial cells in a medium containing 35 mM glucose for 24 h to establish an in vitro model of HG-induced pyroptosis. CCK-8 experiments showed reduced cell proliferation, indicating cell death induced by HG. Carvedilol intervention promoted myocardial cell proliferation.

ROS and oxidative stress are closely associated with diabetes and its complications [37]. In the development of Type 2 diabetes, elevated insulin levels increase ROS levels, activate NLRP3 inflammasomes, and trigger subsequent inflammatory responses [38–40]. Under prolonged HG conditions, cardiac cells release more ROS from mitochondria, leading to NLRP3 inflammasome activation [41]. As a result, SREBP2 and its downstream effector molecule NF-*κ*B are activated, leading to the interaction between NLRP3 and the CARD-containing apoptosis-associated speck-like protein, ultimately activating the inflammasome [41]. Activated NLRP3 inflammasomes cleave pro-caspase-1 into active caspase-1, inducing pyroptosis in mouse cardiac cells, resulting in the release of IL-1*β*, IL-18, and chemokines, activating matrix metalloproteinases and other proteases, exacerbating inflammation and myocardial damage [41, 42]. In this study, HG stimulation increased ROS production in myocardial cells, which was reduced relative to the intervention of carvedilol, suggesting that carvedilol can alleviate HG-induced myocardial cell damage.

NLRP3 inflammasomes are multiprotein complexes that can recognize pathogen-associated molecular patterns or damage-associated molecular patterns and recruit and activate the inflammatory protease caspase-1 [43]. During pyroptosis, activated caspase-1 cleaves pro-IL-1*β* and pro-IL-18 into their mature forms [44]. It also cleaves the N-terminal sequence of GSDMD, forming pores on the cell membrane, disrupting membrane integrity, and releasing cellular contents, leading to cell disintegration and death [45, 46]. Research has confirmed that NLRP3 inflammasomes play an important role in myocardial cell pyroptosis as well as the occurrence and development of Dia-CM [47, 48]. The expression of NLRP3 inflammasomes is higher in cultured myocardial cells stimulated by HG [49]. Studies have shown that carvedilol has antioxidant effects and can protect against peritonitis by inhibiting NLRP3 inflammasome activation and macrophage pyroptosis [36]. In this study, the expression of pyroptosis-associated proteins such as NLRP3, pro-caspase-1, GSDMD-N, SREBP2, NF-*κ*B, and p-NF-*κ*B was upregulated in myocardial cells stimulated by HG, but carvedilol significantly mitigated their expression. These results suggest that NLRP3 may be a novel therapeutic target for cell pyroptosis in the clinical treatment of Dia-CM and that carvedilol may exert a protective effect on myocardial cells by inhibiting pyroptosis.

**Figure 5:**
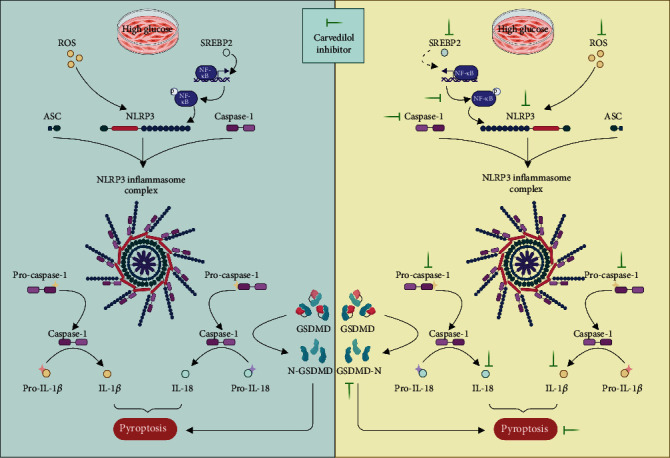
Schematic representation of the mechanism by which carvedilol reduces myocardial cell damage in a high glucose environment by inhibiting NLRP3-ASC inflammasome and caspase-1/GSDMD signaling pathways.

This study delves into the mechanisms of HG environment impact on H9C2 myocardial cells and the regulatory role of carvedilol through transcriptomic analysis and in vitro experiments. Transcriptomics unveiled that cell inflammation and pyroptosis might be pivotal factors leading to Dia-CM. Subsequent in vitro experiments demonstrated that the HG environment significantly decreased the cell viability of H9C2 cells, elevated ROS levels, triggered inflammatory responses, and promoted the overexpression of NLRP3 inflammasome-related proteins. Treatment with carvedilol notably ameliorated these adverse effects, particularly showing optimal efficacy at moderate doses. Furthermore, supplementation with NLRP3 inhibitor (MCC950), caspase-1 inhibitor (VX-765), or ROS inhibitor (NAC) further improved the adverse effects. This suggests that carvedilol may mitigate myocardial cell damage under HG conditions by inhibiting the NLRP3-ASC inflammasome and Caspase-1/GSDMD signaling pathways (Figure 5). While the protective role of carvedilol in diabetic cardiomyopathy has been reported in the literature by Wang et al., the molecular mechanisms by which it exerts its effects in diabetic cardiomyopathy remain undocumented. This study uncovers potential molecular mechanisms through which carvedilol inhibits myocardial cell damage under HG conditions at the molecular and cellular levels, providing new theoretical foundations for Dia-CM prevention and treatment.

This study elucidates the potential mechanism by which carvedilol inhibits myocardial cell damage in a HG environment at the molecular and cellular levels, offering a novel theoretical basis for the prevention and treatment of diabetic cardiomyopathy (Dia-CM). Carvedilol, a commonly used clinical medication, has been widely verified for its safety and effectiveness. Therefore, this research not only enhances our understanding of the pathogenesis of Dia-CM but also presents new strategies for its clinical management.

Although this study has yielded positive results, there are several limitations. Primarily, the research was predominantly conducted at the in vitro level without validation in animal models or clinical samples. Future investigations should confirm the effect of carvedilol in inhibiting myocardial cell damage in a HG environment across a broader range of cell types and animal models to ensure its potential application in Dia-CM therapy. Furthermore, while carvedilol has been shown to inhibit the NLRP3 inflammasome, further research is required to elucidate its specific molecular mechanism. Understanding the precise molecular mechanism by which carvedilol inhibits NLRP3 inflammasome activity will facilitate the development of more precise therapeutic strategies. Studies have indicated that certain clinical drugs can also inhibit the NLRP3 pathway. For instance, salidroside can regulate the NF-*κ*B/NLRP3/caspase-1 signaling pathway to inhibit cell apoptosis and pyroptosis [50]; meanwhile, anthocyanins can suppress cell inflammation and oxidative damage by inhibiting the NLRP3/caspase-1 signaling pathway [51]. These findings suggest that carvedilol may target the NLRP3 pathway through similar molecular mechanisms, which will be a key focus of our future research endeavors. Additionally, this study did not consider other potential influencing factors, such as the duration of diabetes, patient age, and gender, which could impact the interpretation of the research outcomes. Given the individual variations among patients, conducting large-scale clinical trials to assess the safety and efficacy of carvedilol in Dia-CM treatment is a crucial direction for future research. Ultimately, these studies will offer a more robust scientific foundation and enriched clinical experience for the prevention and treatment of Dia-CM.

## 5. Conclusion

Future research should validate the effectiveness of carvedilol in inhibiting myocardial cell damage under HG conditions in a broader range of cell types and animal models to ensure its potential application in Dia-CM treatment. Moreover, exploring the specific molecular mechanisms by which carvedilol inhibits NLRP3 inflammasome activity will facilitate the development of more precise therapeutic strategies. Furthermore, considering the individual variations in diabetic patients, conducting large-scale clinical trials to evaluate the safety and efficacy of carvedilol in Dia-CM treatment is an important direction for future research. Ultimately, these studies will provide a solid scientific foundation and valuable clinical experience for the prevention and treatment of Dia-CM.

## Figures and Tables

**Table 1:** H9C2 cell proliferation assay.

**Groups**	**OD values (** $\bar{x}\pm s$ , **n = 5)**
Blank	0.789 ± 0.039
HG	0.533 ± 0.036△
HG+MCC950	0.654 ± 0.066△▲
HG+VX-765	0.697 ± 0.032△▲
HG+NAC	0.669 ± 0.065△▲

*Note:* △Compared with the blank group, *p* < 0.05. ▲Compared with the HG intervention group, *p* < 0.05.

Abbreviations: HG, high glucose; NAC, N-acetyl-L-cysteine; OD, optical density.

**Table 2:** Analysis of inflammatory cytokines in H9C2 cells from each group.

**Groups**	**ROS fluorescence (%)**	**IL-1*β* (pg/mL)**	**IL-18 (pg/mL)**
Blank	100.000 ± 3.984	13.942 ± 2.792	0.161 ± 0.018
HG	144.892 ± 5.309△	31.494 ± 1.511△	0.353 ± 0.035△
HG+MCC950	126.835 ± 4.919△▲	23.162 ± 3.046△▲	0.247 ± 0.016△▲
HG+VX-765	109.402 ± 1.800△▲▽	19.398 ± 3.349▲	0.207 ± 0.021△▲
HG+NAC	112.427 ± 4.338△▲▽	21.406 ± 3.967△▲	0.238 ± 0.018△▲

*Note:* Each bar/point represents the mean ± SEM (*n* = 3). △Compared with the blank group, *p* < 0.05. ▲Compared with the HG group, *p* < 0.05. ▽Compared with the HG+MCC950 group, *p* < 0.05.

Abbreviations: IL, interleukin; ROS, reactive oxygen species.

**Table 3:** Expression of pyroptosis-related proteins in each group.

**Groups**	**NLRP3**	**ASC**	**Pro-caspase-1**	**GSDMD-N**
Blank	0.188 ± 0.015	0.762 ± 0.063	0.368 ± 0.112	0.208 ± 0.009
HG	0.431 ± 0.009△	0.777 ± 0.027	0.800 ± 0.058△	0.473 ± 0.038△
HG+MCC950	0.293 ± 0.030△▲	0.791 ± 0.021	0.647 ± 0.058△	0.344 ± 0.029△▲
HG+VX-765	0.318 ± 0.079△▲	0.722 ± 0.029	0.469 ± 0.109▲▽	0.288 ± 0.017△▲▽
HG+NAC	0.302 ± 0.045△▲	0.760 ± 0.036	0.485 ± 0.093▲	0.296 ± 0.042△▲

*Note:* Each bar/point represents the mean ± SEM (*n* = 3). △Compared with the blank group, *p* < 0.05. ▲Compared with the model group, *p* < 0.05. ▽Compared with the HG+MCC950 group, *p* < 0.05.

Abbreviations: ASC, apoptosis-associated speck-like protein containing a CARD; GSDMD, gasdermin D; NLRP3, nucleotide-binding domain, leucine-rich-containing family, pyrin domain-containing-3.

**Table 4:** Analysis of inflammatory cytokines secreted by H9C2 cells from each group.

**Groups**	**OD values**	**IL-1*β* (pg/mL)**	**IL-18 (ng/mL)**	**LDH (U/L)**
Blank	0.763 ± 0.021	11.388 ± 2.852	0.153 ± 0.017	83.333 ± 14.869
Model	0.514 ± 0.033△	29.780 ± 3.110△	0.352 ± 0.017△	233.333 ± 18.898△
Model+CVL low-dose	0.633 ± 0.026△▲	22.896 ± 1.962△▲	0.236 ± 0.024△▲	189.682 ± 34.200△▲
Model+CVL medium-dose	0.665 ± 0.029△▲	17.286 ± 1.651△▲▽	0.214 ± 0.026△▲	111.111 ± 31.437▲
Model+CVL high-dose	0.619 ± 0.037△▲▼	24.950 ± 2.620△▲▼	0.244 ± 0.019△▲	143.651 ± 11.252△▲

*Note:* Each bar/point represents the mean ± SEM (*n* = 3). △Compared with the blank group, *p* < 0.05. ▲Compared with the high-glucose model group, *p* < 0.05. ▽Compared with the model+CVL low-dose group, *p* < 0.05. ▼Compared with the model+CVL medium-dose group, *p* < 0.05.

Abbreviations: CVL, carvedilol; LDH, lactate dehydrogenase.

**Table 5:** Analysis of ROS levels in H9C2 cells from each group.

**Groups**	**ROS fluorescence (%)**
Blank	100.000 ± 2.635
High-glucose model	149.898 ± 4.000△
Model+CVL	106.036 ± 2.211▲
Model+CVL+MCC950	85.012 ± 1.211■
Model+CVL+VX-765	87.169 ± 1.065■
Model+CVL+NAC	88.012 ± 1.539■

*Note:* Each bar/point represents the mean ± SEM (*n* = 3). △Compared with the blank group, *p* < 0.05. ▲Compared with the high-glucose model group, *p* < 0.05. ■Compared with the model+CVL group, *p* < 0.05.

Abbreviation: ROS, reactive oxygen species.

**Table 6:** Expression of pyroptosis-related proteins in H9C2 cells from each group.

**Pyroptosis-related proteins**	**Blank group**	**High-glucose model group**	**Model+CVL group**	**Model+CVL+MCC950**	**Model+CVL+VX-765**	**Model+CVL+NAC**
NLRP3	0.339 ± 0.029	0.657 ± 0.043△	0.492 ± 0.027△▲	0.406 ± 0.048■	0.395 ± 0.037■	0.416 ± 0.029■
ASC	0.847 ± 0.022	0.845 ± 0.038	0.853 ± 0.029	0.829 ± 0.041	0.843 ± 0.056	0.831 ± 0.050
Pro-caspase-1	0.359 ± 0.011	0.713 ± 0.019△	0.486 ± 0.005△▲	0.315 ± 0.016■	0.329 ± 0.037■	0.304 ± 0.023■
SREBP2	0.229 ± 0.032	0.592 ± 0.065	0.382 ± 0.046	0.254 ± 0.042	0.272 ± 0.036	0.267 ± 0.043
GSDMD-N	0.332 ± 0.055	0.787 ± 0.120△	0.558 ± 0.128△▲	0.357 ± 0.061■	0.342 ± 0.052■	0.326 ± 0.038■
NF-*κ*B	0.725 ± 0.019	0.716 ± 0.062	0.723 ± 0.047	0.713 ± 0.051	0.731 ± 0.064	0.746 ± 0.059
p-NF-*κ*B	0.501 ± 0.034	0.842 ± 0.079△	0.660 ± 0.089△▲	0.428 ± 0.069■	0.463 ± 0.071■	0.435 ± 0.056■

*Note:* Each bar/point represents the mean ± SEM (*n* = 3). △Compared with the blank group, *p* < 0.05. ▲Compared with the high-glucose model group, *p* < 0.05. ■Compared with the model+CVL group, *p* < 0.05.

Abbreviations: NF-*κ*B, nuclear factor-*κ*B; SREBP2, sterol regulatory element-binding protein 2.

## Data Availability

The data that supports the findings of this study are available on request from the corresponding author.
